# Hearing impairment diagnosis in Pernambuco: services of intermediate complexity - 2003.

**DOI:** 10.1016/S1808-8694(15)31012-0

**Published:** 2015-10-19

**Authors:** Gabriella Morais Duarte Miranda, Bianca Arruda Manchester de Queiroga, Fábio José Delgado Lessa, Mariana de Carvalho Leal, Sílvio da Silva Caldas Neto

**Affiliations:** a MS student in Public Health - CPqAM/FIOCRUZ, Substitute Professor - Federal University of Pernambuco.; b PhD in Psychology - Federal University of Pernambuco, Assistant Professor - Federal University of Pernambuco.; c PhD student in Nutrition - Federal University of Pernambuco, Assistant Professor - Federal University of Pernambuco.; d PhD in Otorhinolaryngology - University of São Paulo. Otorhinolaryngologist - Real Instituto de Otorrinolaringologia do Real Hospital Português de Beneficência em Pernambuco.; e Associate Professor - University of São Paulo - Associate Professor - Federal University of Pernambuco. Federal University of Pernambuco -UFPE.

**Keywords:** Diagnosis, Hearing Impairment, Intermediate Complexity

## Abstract

Universal and equal access to health care, granted to the population as of the brazilian constitution of 1988, made it easier the early diagnosis of hearing impairment. Thus, through the sistema único de saúde (sus), public and private health care providers granted this health coverage to the whole brazilian population, in the different levels of complexity.

**Aim:**

this paper aims at studying the supply of intermediate complexity hearing impairment diagnosis in pernambuco in the first semester of 2003.

**Materials and Methods:**

a study of series analyzed 17669 procedures and 372 professionals in the field of hearing impairment in the 185 municipalities in the state of pernambuco - brazil - data taken from the outpatient information system of the ministry of health.

**Results:**

the results showed that the procedures are being carried out in only five municipalities, most of them managed in municipal units. We also identified an unequal distribution of diagnostic procedures.

**Conclusion:**

we then concluded that much is still necessary to provide hearing impairment diagnostic in a horizontal fashion, considering the community differences through an universal, integrated and efficient model, as the one proposed by the sistema único de saúde.

## INTRODUCTION

With the Sistema Único de Saúde (SUS) - Brazilian Public Health System, access to health care programs and such actions became widespread and equally provided, in other words, the SUS is responsible for providing health care to all people through actions involving health promotion, prevention, protection and recovery. More recently, with the Norma Operacional de Assistência à Saúde - NOAS 01/2001- Health Care Operational Standard, there was a competition among municipalities for the incorporation of more complex and higher cost technology, aiming at increasing health problem solving capability in the various levels of health care. Moreover, it aims at broadening the responsibility of the municipalities in Basic Care - the very definition of the process of regionalization of care - creating mechanisms to strengthen SUS management and to update the capability criteria for states and municipalities. Thus, access to all levels of health care, broadening basic care and guaranteeing reference to the other care levels to bring them increasingly closer to the population.^1^

With widespread and equally distributed access to health care actions (policies and programs) and health promotion, protection and prevention, guaranteed through the Constitution of 1988, the early diagnosis of hearing impairment has become more accessible. Thus, through SUS, led by the public sector and using operational standards that define means of financing, tasks and the competences of each level of the administration, both public and partnering private health care providers should provide, free of charge, this coverage to all the Brazilian Population, at the different levels of complexity.

Hearing impairment early diagnosis is fundamental so as to have the possibility of early intervention in such cases. No isolate test has absolute diagnostic value; the interaction of many tests, together with the medical interview and clinical exam helps in concluding the diagnosis.

Because of the severe consequences to language development and the high incidence of hearing impairment in children, early hearing impairment detection programs have been recommended and developed in hospital nurseries through otoacoustic emissions. This test has become a fundamental tool in the objective assessment of sensorineural hearing impairments.^2^

It is estimated that 42 million people above 3 (three) years of age have some kind of hearing impairment, from moderate to profound.^3^ There was an estimate that the number of hearing loss affected people would come to 57 million in the year 2000.^4^

Approximately 0.1% of children are born with severe to profound hearing loss, and about 90% of these children are offspring of normal hearing parents.^5^ This type of hearing impairment is severe enough to prevent normal language acquisition through hearing. And over 4% of the high risk children are diagnosed as having moderate to profound hearing loss.^6^

It is estimated that in the Brazilian population, 1.5% - about 2,250,000 inhabitants, have some hearing impairment, and this occupies the third place among all the disabilities found in our country.^6^

The health care actions offered by Sistema Único de Saúde (SUS) are characterized according to their complexity. The low complexity action is based on prevention, and it should be offered on the basic health care network; the intermediary complexity involves a set of actions and services in wards and hospitals which aim at caring for the major problems that affect the population, of which clinical practice require the availability of specialized professionals and the use of diagnostic and therapeutic technological resources, and its scope is not justifiable in all cities of the country. And the high complexity, requiring high technology and high cost services.

As far as audiology is concerned, basic care actions are not performed by the otorhinolaryngologist, or by the speech and hearing therapist, because they are not part of the family health care team that focus on this level of care. The procedures present in the list of Outpatient Information System from the Ministry of Health does not include hearing health promotion or prevention, not even hearing screening, thus not being performed by physicians, nurses, nurse assistants and community health agents, even under the guidance of a qualified professional. Among the procedures listed in the intermediary complexity list are the preliminary diagnoses, such as tonal and vocal audiometry. In cases of hearing assistance, there is the need of a through follow up, since it is common to have many complementary exams done, besides a hearing aid selection and fitting program together with language rehabilitation. The latter is considered of high complexity because it requires differential diagnosis and high technology intervention, thus raising treatment costs.

Therefore, we need to broaden especially the intermediary complexity list of procedures, increasing coverage, specially for hearing screening of populational groups, such as newborns, school-aged children, noise-exposed workers and elderly patients. Thus, the SUS principles of universality, equity and integrality would be better followed.

## OBJECTIVES

The general goal of the present investigation is to study the supply of Hearing Impairment diagnosis services, considering the intermediary complexity tier in Pernambuco in the 1st semester of 2003. And as specific objective: to describe the intermediary complexity diagnostic procedures in Hearing Impairment according to professional, type of provider, municipality and mode of municipal management.

## MATERIALS AND METHODS

Our investigation studied 2293 health units that serve the 185 cities in the State of Pernambuco, from January to June of 2003.

Our sample included 17669 hearing impairment diagnostic procedures enrolled in the intermediary complexity tier and 372 hearing impairment diagnosis professionals (otorhinolaryngologists and speech and hearing therapists)

We carried out an observational, descriptive, cross-sectional study.

The following variables were considered: type of professional, type of provider, municipality and municipal administration mode.

As data collection instruments, we explored the ambulatorial production archives PAPEAAMM.DBC from January to June of 2003 and of professional activity from the ATPEAAMM files from June of 2003, maintained at the Outpatient Information System at the Ministry of Health. All the files were then processed by the TABWIN software produced by the IT Department of the Ministry of Health; we then had to create definition and conversion files in order to facilitate the creation of tables.

Since these were data available on the Internet, without identification and because they were clustered in Health Units and Municipalities, it was not necessary to present any Informed Consent form, nor Ethics Committee approval, saved for the source disclosure: Outpatient Information System - Ministry of Health

## RESULTS AND DISCUSSION

Graph 1 depicts the hearing impairment diagnostic procedures according to type of provider. With the municipalization process of the health care system, this high production from the municipal public health care provider was expected, having seen that they were present in larger numbers in the health unit register.

To municipalize health care meant to acknowledge the municipality's political responsibility towards the health of its citizens. To carry out such task, it is necessary to gather the different health care resources, making them available under the municipal administration, which starts to formulate local policies, planning, organization, execution, evaluation and control of health care actions and measures locally.^7^ It must be clear that health care goals vary socially and historically; thus, health care models must be revised and updated based on the continuous analysis of the populational health situation.^8^

Table 1 shows the prevalence of procedures that make up basic audiologic evaluation. Such findings point toward a predominance of audiometries, followed by acumetry procedures, and finally, immitanciometry.

**Table 1:** Hearing Impairment Diagnostic Procedures description in the cities of the State of Pernambuco, in the first semester of 2003, according to municipality.

Intermediary complexity procedure	Bezerros	Gravatá	Limoeiro	Petrolândia	Recife	Total
Vocal audiometry. Sound discrimination threshold -sd	0	4	26	2	911	943
Vocal audiometry - intelligibility threshold -sr	123	0	47	0	3148	3318
Vocal audiometry - speech. Recognition threshold -ir	123	0	42	0	2775	2940
Timpanometry	0	0	0	0	890	890
Static compliance /stapedian reflex study	0	0	0	0	823	823
Stapedial reflex decay study	0	0	0	0	333	333
Weber test (tuning fork)	0	0	0	0	2293	2293
Rinne test (tuning fork)	0	0	0	0	257	257
Impedanciometry	124	0	0	3	251	378
Threshold tonal audiometry	126	4	72	5	3862	4069
Detection screened school children with hearing impairment 1f-camp	0	0	0	0	855	855
Others	5	0	1	1	563	570
Total	501	8	188	11	16961	17669

Basic audiologic exams described in the literature are mainly defined by three procedures: tonal audiometry, SRT and immitanciometry. Notwithstanding, acumetry, which encompasses Weber and Rinne tests should be the prevailing one, having in mind that all it takes is a tuning fork. We should expect an equal distribution of both procedures, but that was not observed in our results. With the tuning fork, it is possible to perform both qualitative and quantitative tests, and, through comparative tests between air and bone conduction gain information on the site of lesion: outer, middle or inner ear.^9^ Audiometry should be performed only for complementary purposes, and immitanciometry only for the cases that need it. However, the findings point in another direction, calling our attention to a lack of actions.

It is also important to highlight the fact that the audiometric Weber test is not included on the List of the Outpatient Information System as a diagnostic procedure for Hearing Impairment diagnosis, because this bone conduction test complements tonal audiometry, confirming or not the type of audiometric curve found.

Moreover, we stress the fact that the summation of vocal audiometries should be equivalent to the tonal audiometry, and such fact was also not found, since soon after determining the tonal thresholds by air and bone (tonal) conduction, the audiologist should investigate the level of detection and recognition of oral language by the individual. Vocal audiometry (SRT) provides a direct and global hearing measure for speech, providing information that may help: in the topodiagnosis; in the development of the individual's social-hearing skills; in confirming tonal thresholds; and in indicating a hearing aid for the affected individuals. It is carried out in two stages: (Speech Reception Threshold - SRT) and study of the Speech Recognition Percentage Index. Because of the patient's difficulty of communication, we study the patient's Speech Detection Threshold - SDT.^9^

As to impedanciometry, which consists in the performance of these three measures: tympanometry, static impedance or tympanic membrane compliance and the stapedial muscle reflex, called stapedial reflex^10^ should be computed together, and this also did not happen.

Such findings suggest a supply of procedures that compromise diagnostic quality, because it does not provide the users with a set of procedures that could aid in the conclusion of the cases, or even, the existence of an underestimate of procedures which are performed, but not recorded.

It is worth highlighting the fact that among the procedures classified as Others, we included the Otoacoustic Emissions (OAE); and only 114 procedures were exclusively carried out at the Instituto Materno Infantil de Pernambuco (IMIP). This data highlights the importance OAE has in hearing impairment screening, especially in premature newborn babies. In this hospital, the otoacoustic Emissions Device (OAE) is used only for the Mãe Canguru Project children, which aims at low birth weight babies and those that are using antibiotics and have to remain in the maternity until the end of treatment. It is estimated that about 800 premature babies are born at IMIP every semester, and all of them should undergo this hearing screening. We noticed that only 114 procedures were carried out, showing the low coverage of the exam towards the target population.

As we saw on Table 1, only five cities offer the hearing impairment diagnostic procedures, thus, it is relevant to present Chart 1, with the distribution of the cities in the State of Pernambuco, in the first semester of 2003, according to the municipal management modes determined by the Basic Operational Standard/1996.

**Chart 1:**
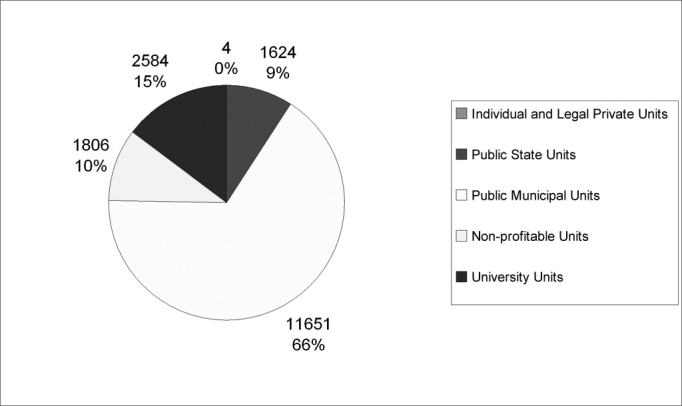
Distribution of hearing impairment diagnostic procedures in the cities of Pernambuco State, in the first semester of 2003, according to service provider type.

**Source:** Ministry of Health / Outpatient Information System.

With the 1996 Basic Operational Standard, there has been a change in the SUS local management modes, which became two: Full in basic health care and full for the health care system. In the first mode, the municipalities started to be fully responsible for outpatient care, for the basic initiatives of sanitary and epidemiologic surveillance and for the management of all the basic health care units in the region. In the latter, besides basic care, they also became responsible for all the initiatives regarding the Sistema Único de Saúde services in their area of responsibility, including the offer of intermediate and high technology complexity.^11^

**Chart 1:**
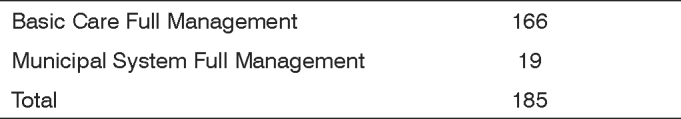
Distribution of cities in the State of Pernambuco, in the first semester of 2003, according to municipal management modes, determined by the Basic Operational Standard 01/96.

**Chart 2:**
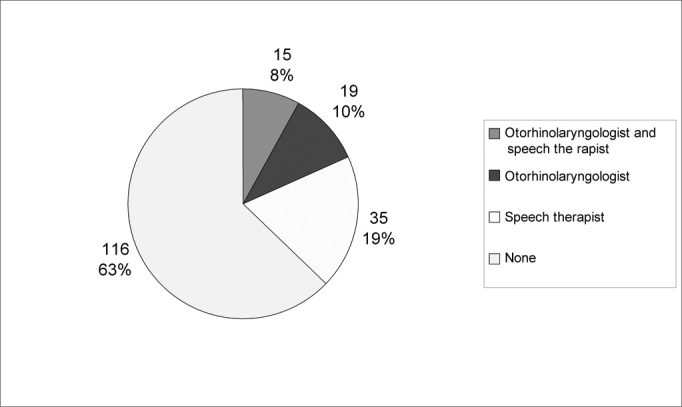
Distribution of hearing impairment diagnosis specialists in the State of Pernambuco, in the first semester of 2003, according to city.

**Source:** Ministry of Health / Outpatient Information System.

This means that 19 municipalities should offer hearing impairment diagnostic procedures, however, only 3 of these are offering this type of service to the population. Besides these, 2 cities, certified for Basic Health Care Full Management also offer hearing diagnosis. This aspect shows that the goal of municipalization is to allow the municipality to take over and act as a base for the Federation^12^, for some reason - due to difficulties in resource allocation or lack of infrastructure, is not being fulfilled.

Graph 2 shows the distribution of hearing impairment diagnostic professionals in the cities of the State of Pernambuco, and it is possible to see that 63% of the municipalities do not have any professional in this category.

Of the 15 cities that have an otorhinolaryngologist and a hearing and speech specialist, only two offer hearing impairment diagnostic procedures. This highlights some problems. We see that the cities are highly heterogenous, some are too small to manage a complete functional system in their territory, and other that require more than one system in their area, and they are, simultaneously regional attraction poles.^13^

We must consider the differences among the many cities that make up the state, pertaining to size, population and social and economic differences, which interfere in health care structuring, mainly if we consider that health care levels bear different technological make up related to its cost, density and feasibility.^14^

Notwithstanding, we acknowledge that there is a generalized effort of public management for the planning of actions that meet the requirements of the population, and with that, access to health care services will be available without restrictions and discriminations.

## CONCLUSION

Through this investigation we realized that hearing impairment diagnostic procedures are available in only five cities of the State of Pernambuco, while at least 19 of them, responsible for intermediate complexity procedures coverage, should guarantee access to such procedures and services.

Data also show that the municipal public provider was responsible for 66% of the procedures, and such fact shows the strong power of this municipalization proposed by the Sistema Único de Saúde.

We have also observed that 63% of the municipalities do not have any hearing impairment diagnostic professional, being those otorhinolaryngologists or audiologists, duly registered in the Outpatient Information System from the Ministry of Health and, therefore, these cities do not offer their population any action, even in basic health care, that promote hearing health.

Much of the data have suggested a possible under-recording of actions, not meeting the offer capability of the service-rendering entities, thus compromising the quality of services provided, and thus, it is imperative to reorganize it in order to maximize the use of the available resources.

New studies are being performed in order to analyze the offer of these procedures in all the country. Notwithstanding, the data hereby presented suggest that there is still much to be done in our State, so that access to hearing impairment diagnostic procedures may serve the needs of each community, in a horizontal fashion, respecting their differences, within an equal, integrated, sustainable and, most specially, efficient model.
